# Human cytomegalovirus-encoded microRNAs expression profile in plasma of patients with aortic dissection

**DOI:** 10.1186/s13019-023-02122-7

**Published:** 2023-01-18

**Authors:** Yongqing Cheng, Yufan Du, Qi Wang, Qinghe Lv, Yunxin Xue, Weihong Zhou, Chenyu Zhang, Xi Chen, Dongjin Wang

**Affiliations:** 1 Department of Cardio-Thoracic Surgery, Drum Tower Hospital Affiliated to Nanjing University Medical School, Nanjing, 210008 Jiangsu China; 2 Present Address: State Key Laboratory of Pharmaceutical Biotechnology, Collaborative Innovation Center of Chemistry for Life Sciences, Jiangsu Engineering Research Center for MicroRNA Biology and Biotechnology, NJU Advanced Institute for Life Sciences (NAILS), School of Life Sciences, Nanjing University, Nanjing, 210033 Jiangsu China; 3 Department of Health Management Centre, Drum Tower Hospital Affiliated to Nanjing University Medical School, Nanjing, 210008 Jiangsu China

**Keywords:** Human cytomegalovirus, MicroRNA, Plasma, Aortic dissection

## Abstract

**Background:**

Aortic dissection (AD) is a rare disease with high mortality for which no effective diagnostic biomarkers are available. Human cytomegalovirus (HCMV) infection is an important cause of the occurrence and progression of many diseases, but the relationship between HCMV infection and AD is not clear.

**Methods:**

In this study, we first used quantitative reverse transcription polymerase chain reaction (qRT-PCR) to determine the expression profile of 25 HCMV-encoded microRNAs (HCMV miRNAs) in the plasma within a training set consisting of 20 AD patients and 20 healthy controls. Then, abnormal expressed HCMV miRNAs were verified in a validation set of 12 AD patients and 12 healthy controls. In addition, HCMV infection was detected in the third cohort consisting of 20 AD patients and 20 healthy controls.

**Results:**

The 95% quantile of the expression levels of HCMV miRNAs in the training set was used as the threshold for distinction between AD patients and healthy controls. The proportion of individuals with high level of five types of HCMV miRNAs was significantly different between AD patients and healthy controls. In the validation set, only the proportion of individuals with high levels of hcmv-miR-UL112-5p and hcmv-miR-UL22A-5p, two of the five HCMV miRNAs obtained in the preliminary screening, showed significant difference between AD patients and healthy controls. In the third cohort, there was no significant difference in HCMV DNA levels and anti-HCMV IgG concentrations between AD patients and healthy controls.

**Conclusions:**

The HCMV miRNAs levels in plasma differed in AD patients and healthy controls. This finding may contribute to a further understanding of the relationship between HCMV infection and AD and are worthy of future research on the diagnosis and etiology of AD.

## Introduction

Aortic dissection (AD) occurs when the aortic intima ruptures due to pathological factors. Blood enters the media through the rupture, and the media is dissected into inner and outer layers to form a false lumen, which looks like the dissection in the aortic wall and is enlarged in a “tumor-like” way [1]. Although AD is not a common disease, it has a high mortality rate [2]. The typical symptoms of AD are tearing pain [2, 3] and misdiagnosis occurs in some patients on initial evaluation with atypical symptoms similar to stroke and other disorders, the early diagnosis of AD is very important. However, the current clinical diagnosis of AD appears to be insufficient, and the main diagnostic methods including computed tomography scanning, transthoracic echocardiography, transesophageal echocardiography and magnetic resonance imaging have certain limitations [4, 5]. Therefore, it is necessary to exploit a new method for early and rapid diagnosis of AD and thereby to clarify the pathological mechanism of this disease.

MicroRNAs (miRNAs) serve as one kind of small regulatory RNAs mainly distributed in plasma, saliva and other body fluids [6]. In general, miRNA inhibits the translation of related proteins by complementing one site of the 3′- untranslated region (3′ UTR) of its targeted mRNA to degrade the mRNA or block the translation mechanism of the mRNA [7]. Accordingly, miRNAs participate in many biological processes by regulating protein-coding genes and also associated with many diseases such as allergic diseases and cancer [8, 9]. Considering that individual miRNAs lacks disease specificity while specific combinations of miRNAs are associated with certain disease [10], the alterations of miRNA expressions are hence of great significance in the diagnosis and treatment of diseases [11]. Particularly, previous studies have revealed that a variety of miRNAs derived from the host itself and pathogenic microbes are highly stable in plasma and serum, and that the alterations of circulating miRNA levels in plasma and serum may serve as noninvasive biomarkers for various diseases [12–15].

Human Cytomegalovirus (HCMV) belongs to the herpes virus family, and its genome is the largest in the family, which can encode more than 200 kinds of proteins [16]. Approximately 40% to 99% of the population has been infected with this virus [17]. The newborn and immunosuppressed people infected by this virus may experience symptoms; on the contrary, immunocompetent individuals could avoid those symptoms [18]. The co-existence with host cells or lysis of the host cell is controlled by HCMV-encoded miRNAs [19]. To date, 26 mature HCMV miRNAs have been identified [20]. HCMV miRNAs play a vital role in inhibiting natural killer cells (NK cells) and cytotoxic T cells, as well as in suppressing immune responses such as inflammatory response [21]. Previous researches have shown that changes in the expression profile of HCMV miRNAs can be applied to the disease diagnosis [22, 23]. More importantly, HCMV miRNAs have been confirmed to exist in serum and plasma, and their alterations in serum and plasma are tightly associated with hypertension, hepatitis B and hepatitis C [24–26]. However, the plasma signature of HCMV-encoded miRNAs in AD and its clinical relevance have not been studied.

In this study, we investigated whether HCMV-encoded miRNAs in plasma could be employed as a safe, stable and specific biomarker for AD monitoring. A total of 20 AD patients were enrolled and assigned to the patient group and the same number of healthy individuals served as the control group. After extraction of plasma miRNAs, the miRNA expression profile was detected and the significantly different miRNAs were screened by quantitative reverse transcription polymerase chain reaction (qRT-PCR). Another 12 patients and 12 healthy individuals were subsequently selected for population validation. Statistical analysis was performed using the threshold of preliminary screening to evaluate the clinical value of HCMV miRNA in the diagnosis of AD.

## Patients and methods

### Patient selection

A total of 20 acute AD patients (Stanford type A) were selected as subjects and were diagnosed at the Department of Cardio-Thoracic Surgery of the Affiliated Drum Tower Hospital of Nanjing University Medical School from July, 2016 to July, 2017. In addition, the recruitment of 20 subjects to the parallel control group was conducted in the Department of Health Management Centre of the Affiliated Drum Tower Hospital of Nanjing University Medical School. The clinical and grouping information in detail was shown in Table 1. Participants were divided into a training set consisting of 10 patients and 10 healthy individuals, and the validation set consisting of the remaining 10 patients and 10 healthy individuals. All procedures performed in this study involving human participants were in accordance with the Declaration of Helsinki (as revised in 2013).

**Table 1:** Demographic features of AD patients and healthy controls

Variable	Training set			Validation set			HCMV titers set		
	Case	Normal	*P* value	Case	Normal	*P* value	Case	Normal	*P* value
Number	10	10		10	10		12	12	
Age, years^a^	57.3 ± 13.45	50.8 ± 4.07	0.18^b^	52.7 ± 12.07	52.2 ± 2.78	0.90^b^	48.3 ± 10.76	29 ± 5.95	0.36^b^
Sex, n									
Male	4 (40%)	1 (10%)	0.74^c^	8 (80%)	6 (60%)	0.73^c^	10 (83.3%)	4 (33.3%)	0.98^c^
Female	6 (60%)	9 (90%)		2 (20%)	4 (40%)		2 (16.7%)	8 (66.7%)	

^a^Age data are presented as the mean ± SD, ^b^Student t test_,_^c^Two-sided χ2 test

*AD* aortic dissection, *HCMV* Human cytomegalovirus

This study was approved by Ethics Committee of Drum Tower Hospital Affiliated to Nanjing University Medical School (number of the ethics approval: 2016-197-01) and informed consent was taken from all the participants.

### Blood sample collection and RNA isolation

The whole blood of each participant was collected by venipuncture and then transferred into EDTA-treated centrifuge tube. The blood sample was centrifuged under 4 °C at 800 g for 10 min to separate the plasma and cell fragments. Thereafter, the plasma was further centrifuged under 4 °C at 12,000 g for 10 min to remove a small amount of cell debris. The collecting plasma sample were stored at − 80 °C and transported in a dry ice environment.

Total RNA was extracted from 100 μL plasma samples by phenol/chloroform purification method. Specifically, 300 μL deionized water, 200 μL phenol, and 200 μL chloroform was added to 100 μL plasma and vigorously mixed by vortex. The mixture was incubated at 20 °C for 15 min to segregate the organic phase from the water phase, which was further mixed with 1.5 volume of isopropyl alcohol and 0.1 volume of 3 mol/L sodium acetate (pH 5.3) and stored at − 20 °C for 1 h. RNA pellet was then collected by centrifugation at 16000 g for 20 min. The final product was washed once with 75% ethanol and dried at room temperature for 10 min and then was dissolved in 25 RNase-free water and stored at − 80 °C.

### qRT-PCR analysis

**Fig. 1:**
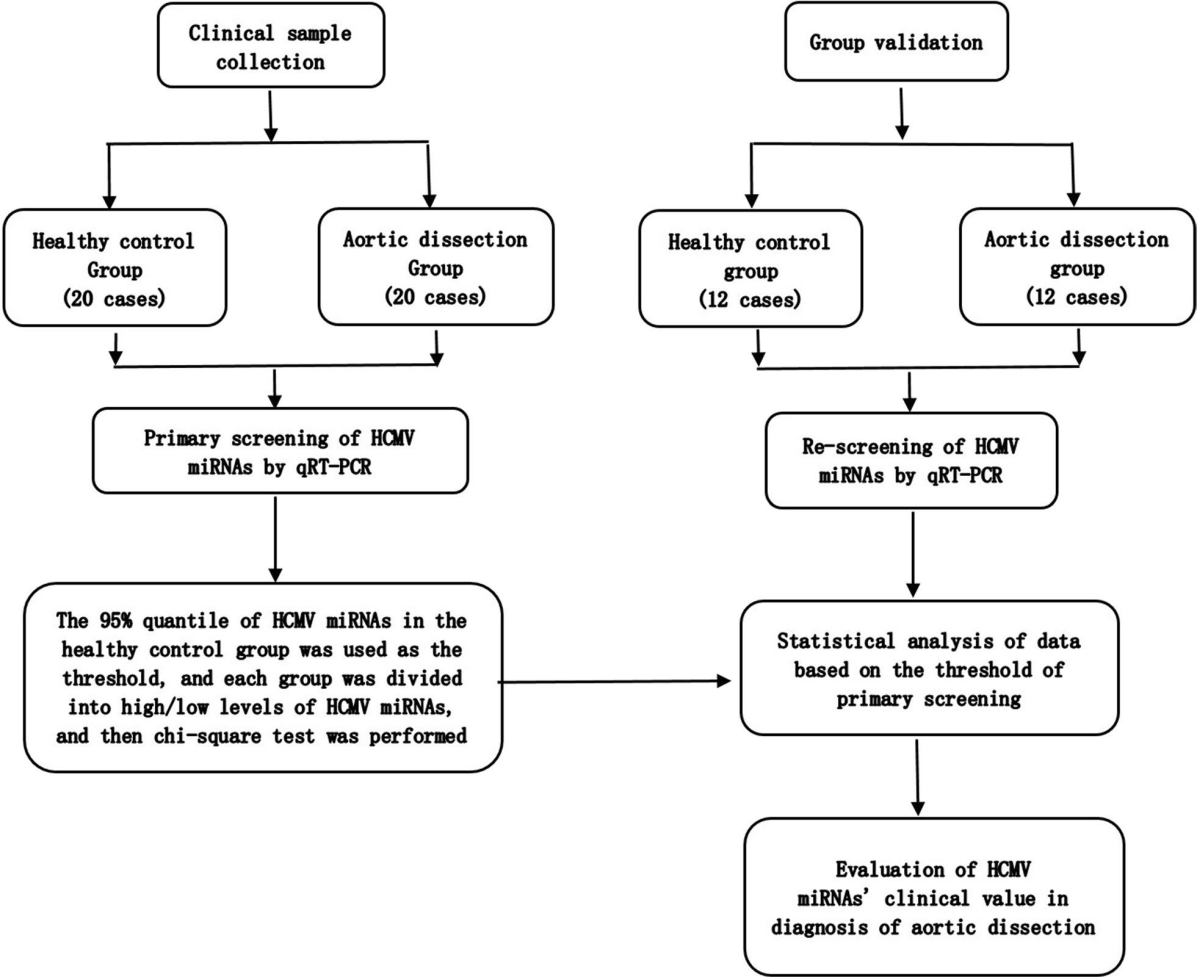
Overview of the study design. *HCMV* Human cytomegalovirus, *miRNA* microRNA, *qRT-PCR* quantitative reverse transcription polymerase chain reaction

We performed a TaqMan probe-based qRT-PCR assay (Fig. 1) to investigate the differential expression of HCMV miRNAs between healthy control group and AD group as described previously [27].

According to the manufacturer's directions, we conducted TaqMan prob-based qRT-PCR to analyse the different expression of HCMV miRNAs between AD patients and healthy controls. First, the dynamic range and sensitivity of the qRT–PCR assays for measuring HCMV miRNAs was determined. Second, a no-template was assessed simultaneously to determine the background noise of the qRT–PCR assays. Only when the CT values of HCMV miRNAs were above the lower boundary of the detection spectrum and when the no-template control was not amplified, the HCMV miRNAs were confirmed to be accurately determined with high consistency. MIR2911 was used as housekeeping genes to standardize the expression of miRNA, and its expression levels did not differ markedly between the two groups. RT primers and PCR primers (Applied Biosystems, Foster City, CA, USA) have high specificity for every target HCMV miRNA. All processes, including non-template controls, and all reactions were performed in triplicate. The relative amount of miRNAs was calculated by 2^−∆Cq^ method.

### ELISA analysis to check anti-HCMV antibodies IgG and IgM in plasma

According to the instructions, HCMV IgG/IgM kit (MEDSON, NJ, USA) was used to detect anti-HCMV IgG and IgM in plasma by ELISA test. For IgG-ELISA, IgG antibody concentrations in each sample were quantified using a standard curve calibrated with the WHO First International standard. For detection of IGM-ELISA, the optical density (OD) value at 450 nm was used to calculate the detection results, and the cut-off value of positive results was OD > 1.2.

### HCMV DNA titers

**Fig. 2:**
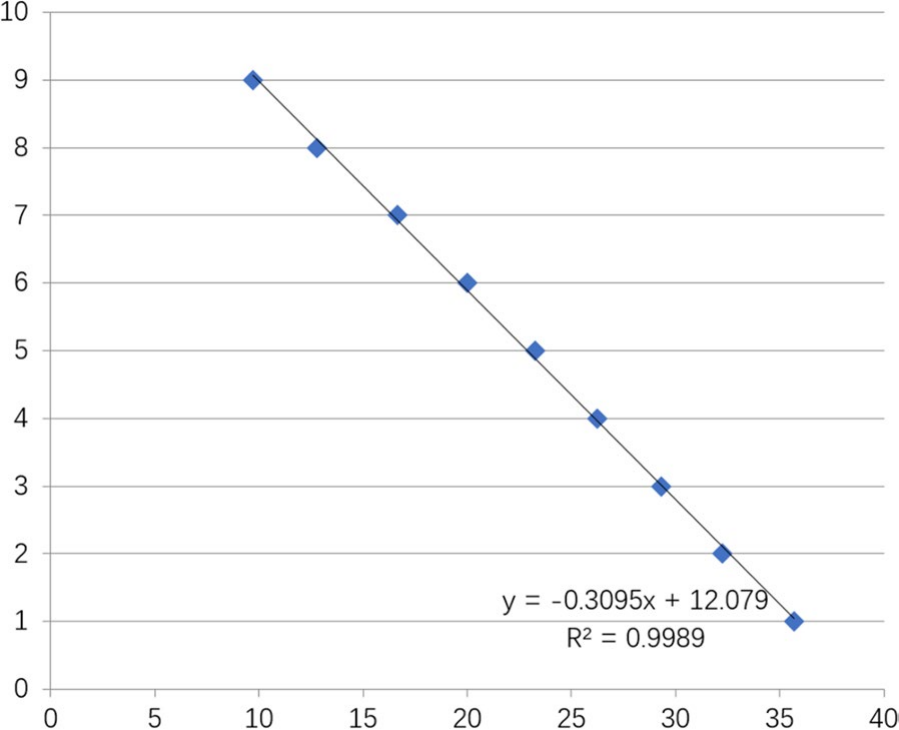
Standard curve of recombinant plasmid that contained the HCMV target sequence

Quantitative PCR was applied to detect the copy number of HCMV in plasma of 12 patients and 12 healthy controls. DNA was extracted from the PBLs according to the protocol of the QIAamp DNA Mini kit (Qiagen, Hilden, Germany). HCMV DNA was detected by using SYBR Green and the following HCMV specific primers: HCMV DNA forward: 5′-CACGG TCCCGGTTTAGCA-3′, HCMV DNA reverse: 5′-CG TAACGTGGACCTGACGTTT-3′. A tenfold dilution of the recombinant plasmid containing the HCMV target sequence was used as the basis for standard curve preparation (Fig. 2). The C_q_ value is converted from the standard curve to the absolute value. The two-step thermal cycle program consists of 45 cycles, denaturation at 95 °C for 15 s, annealing and elongation at 60 °C for 60 s. The results were shown as copies per 1 mL of blood.

### Statistical analysis

Statistical analysis was performed by using SPSS software (19.0 version). Data were expressed as mean ± SD of variables. Chi-square test and two-sided student T test were conducted to analyze the differences of variables between the two groups. Chi-square test is carried out with 95% quantile of training set as cut-off value. Linear regression and logistic regression analyses were used to obtain the equation of the standard curve of recombinant plasmid of HCMV target sequence and to discover the association between plasma HCMV miRNAs and AD, respectively. *P* < 0.05 was considered statistically significant.

## Results

### Determination of the expression profiles of HCMV miRNAs in training set using qRT-PCR analysis

In the preliminary screening, we analyzed HCMV miRNAs in plasma of 20 AD patients and 20 healthy controls using qRT-PCR method and 25 kinds of HCMV miRNAs were detected in both groups (Table 2). Compared with healthy control subjects, AD patients showed higher levels of 5 kinds of HCMV miRNAs, namely hcmv-miR-UL112-5p, hcmv-miR-US25-2-3p, hcmv-miR-US25-2-5p, hcmv-miR-UL69 and hcmv-miR-UL22A-5p (Table 2). The results indicate that the expression levels of specific HCMV miRNAs could be distinguish between AD patients and healthy control subjects.

**Table 2:** Expression profile of HCMV miRNAs in the training set

HCMV encoded miRNAs	95th percentile	High level proportion in control	High level proportion in case	*p* value
hcmv-miR-UL112-5p	228.288	1/20	8/20	0.0197
hcmv-miR-US25-2-3p	233.562	1/20	8/20	0.0197
hcmv-miR-US25-2-5p	162.100	1/20	8/20	0.0197
hcmv-miR-UL69	247.559	1/20	7/20	0.0436
hcmv-miR-UL22A-5p	316.437	1/20	5/20	0.0436
hcmv-miR-US22-5p	3867.293	1/20	6/20	0.0915
hcmv-miR-UL36-3p	97.349	1/20	6/20	0.0915
hcmv-miR-US4-3p	341.574	1/20	6/20	0.0915
hcmv-miR-US5-2-5p	1085.539	1/20	4/20	0.3416
hcmv-miR-UL22A-3p	8.707	1/10	2/10	> 0.999
hcmv-miR-UL36-5p	85,348.030	1/10	0/10	> 0.999
hcmv-miR-UL112-3p	932.203	1/10	2/10	> 0.999
hcmv-miR-UL148D	36,131.795	1/10	2/10	> 0.999
hcmv-miR-US22-3p	160.586	1/10	2/10	> 0.999
hcmv-miR-US29-5p	11,640.902	1/10	1/10	> 0.999
hcmv-miR-US33-5p	248.085	1/10	3/10	0.582
hcmv-miR-UL70-5p	717.324	1/10	1/10	> 0.999
hcmv-miR-UL59	659.824	1/10	1/10	> 0.999
hcmv-miR-US4-5p	15,799.428	1/10	1/10	> 0.999
hcmv-miR-US5-2-3p	5.499	1/10	5/10	0.1409
hcmv-miR-US25-1-3p	245.174	1/10	2/10	> 0.999
hcmv-miR-US29-3p	25.082	1/10	1/10	> 0.999
hcmv-miR-US5-1	445.270	1/10	4/10	0.3034
hcmv-miR-US25-1-5p	16,908.002	1/10	0/10	> 0.999
hcmv-miR-US33-3p	48.292	1/10	4/10	0.3034

*HCMV* Human cytomegalovirus, *miRNA* microRNA

### Confirmation of abnormal expressed HCMV miRNAs in validation set

In the validation set, we further analyzed those five HCMV miRNAs obtained from the preliminary screening in a second cohort consisting of 10 AD subjects and 10 healthy control subjects. The results showed that levels of hcmv-miR-UL112-5p and hcmv-miR-UL22A-5p in plasma had significant difference between two groups. In validation set, the relative expression levels of two selected miRNAs in 20 AD patients and 20 healthy subjects were distinct as shown in Table 3.

**Table 3:** Expression profile of HCMV miRNAs in the validation set

HCMV encoded miRNAs	95th percentile	High level proportion in control	High level proportion in case	*p* value
hcmv-miR-UL112-5p	228.288	0/12	5/12	0.0373
hcmv-miR-UL22A-5p	316.437	0/12	5/12	0.0373
hcmv-miR-US25-2-3p	233.562	12/12	8/12	0.0932
hcmv-miR-US25-2-5p	162.100	7/12	4/12	0.4136
hcmv-miR-UL69	247.559	0/12	1/12	> 0.9999

*HCMV* Human cytomegalovirus, *miRNA* microRNA

### Detection of HCMV DNA and anti-HCMV IgG and IgM in AD patients and healthy controls

**Fig. 3:**
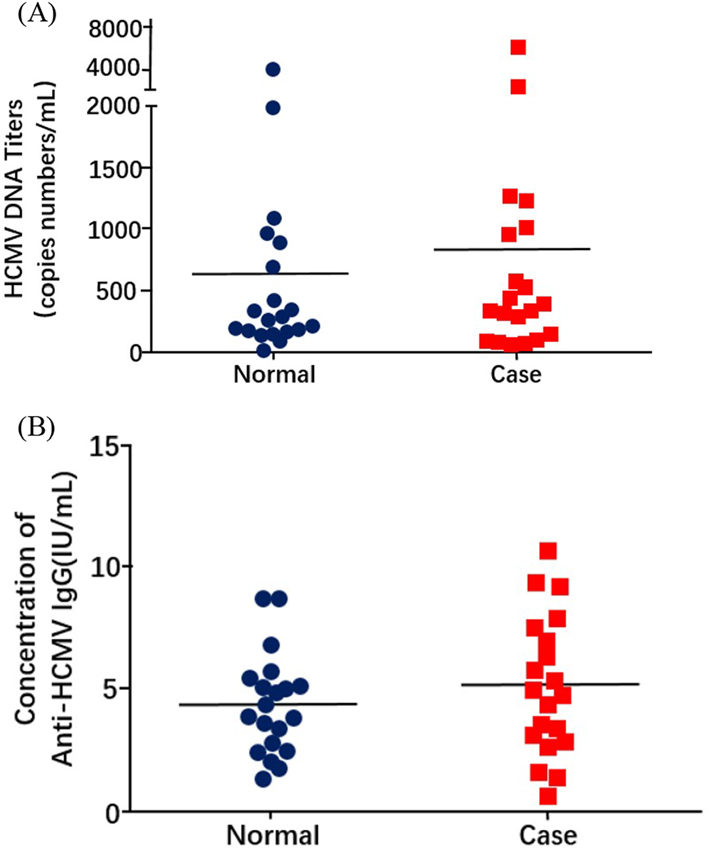
Detection of HCMV and its relationship with HCMV-encoded miRNAs. **A** The HCMV DNA titers were calculated in PBLs of AD patients and healthy controls. **B** Comparison of the concentrations of anti-HCMV IgG in the AD patients (n = 20) versus the healthy controls (n = 20)

Due to the distinct expression levels of HCMV miRNAs in AD patients, we compared HCMV infection in the third cohort containing 20 AD patients and 20 healthy subjects. HCMV DNA in peripheral white blood cell of these two groups was quantitatively detected by PCR method. There was no significant difference of HCMV DNA levels between AD patients and healthy subjects, with the concentrations being 834.4 copies/mL in AD patients and 632.4 copies/mL in healthy subjects, respectively (Fig. 3a). In addition, ELISA analysis was performed to measure concentrations of anti-HCMV IgG and IgM in both two groups. The HCMV antibody test revealed that both two groups were IgG-positive and IgM-negative, and there was no difference of antibody concentrations between two groups (Fig. 3b).

## Discussion

Human cytomegalovirus (HCMV) acts as a widespread pathogen [28]. HCMV infection is usually persistent due to its chronic and latent states of infection [29]. HCMV can be latent for life-long time by replicating at subclinical levels and more importantly it can be reactivated from a latent state in response to normal biology processes [30]. Healthy people infected with HCMV normally have no symptoms, while HCMV infection could put immunocompromised people at risk of severe diseases [31]. HCMV gene products can regulate the host immune response, inhibit the function of some immune cells, and regulate some signaling pathways, such as NF-κB signaling [32, 33]. HCMV miRNAs play a vital role in viral infection and may also be relevant to some diseases of unknown origin [34]. For instance, Li et al. [22] found that highly upregulated hcmv-miR-UL112-3p was associated with essential hypertension. In addition, Liang et al. [35, 36] suggested that hcmv-miR-UL112-3p may also be involved in the pathogenesis of glioblastoma and it could directly target TUSC3 in glioblastoma to exert its oncogene function. In another research, the levels of hcmv-miR-US25-1-5p and hcmv-miR-UL112-3p in extracellular vesicles were found to be relevant to liver damage in infants with HCMV active infection [37]. Pan et al. [38] demonstrated that the levels of unique HCMV miRNAs in chronic hepatitis B patients could be served as a novel biomarker to predict the efficacy of IFNα treatment for chronic hepatitis B patients. Thus, HCMV miRNAs have the potential as a biomarker for diagnosing certain illnesses.

We examined the infection of HCMV in AD patients and healthy control subjects. The results suggested that there was no difference in the load of the latent HCMV genomes between AD patients and healthy controls. Specifically, there was no obvious distinction of HCMV DNA in PBLs between the two groups and both of groups were IgG positive and IgM negative. Based on the detection of HCMV miRNAs in plasma of AD patients and healthy controls, we found that the 25 types of HCMV miRNAs were detected in both of the two groups. Notably, significant difference was observed for the proportion of individuals with above threshold expression level of two types of HCMV miRNA i.e. hcmv-miR-UL112-5p and hcmv-miR-UL22A-5p between AD patients and health controls.

In this study, we discovered that the expression levels of HCMV miRNAs in plasma was highly upregulated in AD patients when compared with healthy controls, based on which the expression profile of HCMV miRNAs may have clinical value in the diagnosis of AD. However, no difference in the load of the latent HCMV genomes between AD patients and healthy controls was observed, which may attribute to the insufficient accuracy of the determination for the infection of HCMV and its state in the host. To sum up, we suggested that HCMV might be associated with the occurrence of AD and further research is needed, especially for the accurate determination of the latent load of HCMV.

Two types of HCMV miRNA i.e. hcmv-miR-UL112-5p and hcmv-miR-UL22A-5p were identified in our study. Previous research revealed that inhibited one of the targets of hcmv-miR-UL112-5p named endoplasmic reticulum aminopeptidase 1 (ERAP1) contributing to the immune escape of HCMV [39]. In addition, detection of the expression of hcmv-miR-UL22A-5p could predict the recurrence of HCMV viremia after discontinuation of antiviral therapy [40]. The above mentioned research figured out the relevance between two types of HCMV miRNAs and diseases associated with HCMV infection. According to this study, we speculated that these two types of HCMV miRNAs might play the same pivotal role in the pathological process in AD and in HCMV infection-related diseases. Considering that the targets of each miRNA are not unique and lack of complete cognition, great efforts should be put into clarifying the function of HCMV miRNAs in the infection progress and revealing the relevance between HCMV miRNAs and diseases.

### Strength and limitations

This study preliminarily proved the clinical application value of HCMV miRNAs expression profile in the diagnosis of AD. In the future, we plan to expand the sample size and carry out multi-center and multi-ethnic group verification to ensure the universality of our results. Finally, in order to apply our results to the actual diagnosis, prospective clinical studies should also be conducted to verify the accuracy and safety of the detection method.

## Conclusion

In summary, there is a significant difference in the proportion of individuals with above threshold expression level of two types of HCMV miRNA between the healthy controls and AD patients. It is necessary to fully understand the relationship between the expression pattern of HCMV miRNAs in vivo and AD before the results can be widely used in clinical practice.

## Data Availability

All raw data are available upon request and the corresponding author, Prof. Dongjin, Wang (E-mail: dongjin_wang@126.com) should be contacted to request the data.

## Abbreviations

AD: Aortic dissection
HCMV: Human cytomegalovirus
miRNA: MicroRNA
HCMV miRNAs: HCMV-encoded microRNAs
3′ UTR: 3′ Untranslated regions
qRT-PCR: Quantitative reverse transcription polymerase chain reaction
ELISA: Enzyme-linked immunosorbent assay
